# Occlusion, TMDs, and dental education

**DOI:** 10.1186/1746-160X-3-1

**Published:** 2007-01-03

**Authors:** Major M Ash

**Affiliations:** 1 Professor and research scientist, emeritus, University of Michigan, USA

## Abstract

The paradigmatic shift to evidence-based dentistry (EBD) that relates to occlusal therapy, selective occlusal adjustment (OA) and stabilization splints therapy (SS) for TMDs has had an unfavourable impact on the teaching of many of the important aspects of occlusion needed in dental practice. The teaching of OA systematically in dental schools has been nearly abandoned because of the belief that OA is an irreversible procedure and gives the impression that it is without merit elsewhere in the management of occlusion. However, a particular dose of knowledge and practice of occlusion that is necessary for all aspects of dental care should be taught systematically in dental schools. The uses and misuses of OA and SS and their limitations should be emphasized because of their importance to bring clinical reality into the dental curriculum. Thus, and irrespective of EBD induced contradictions, OA and SS should still have a significant place in systematically teaching of occlusal therapy. However, there are many more aspects of the management of occlusion that should to be considered. Hopefully, because of their importance, other aspects of the management of occlusion will once again become a significant part of the dental curriculum.

## Review

Quintilian, a Latin rhetorician of the first century, proposed the following advice: Write not so you can be understood but so that you cannot be misunderstood." Hopefully, the following is written so that it is not only understood but also not misunderstood. The following thoughts on occlusal adjustment and the use of stabilization occlusal bite plane splints relate to some of the aspects of occlusal therapy that should be taught systematically in the dental curriculum. The term systematic refers to structured courses with goal oriented outcomes.

### Evidence-based paradigms

The paradigmatic trends in academic dental health sciences reflect attempts to provide a knowledge nexus between the biological and mechanical, or the art and the science aspects of dentistry using probabilistic scientific data. However, the paradigmatic shift to evidence-based dentistry that relates to occlusal therapy, selective occlusal adjustment (OA) and stabilization splints therapy (SS) for TMDs has had an unfavorable impact on the teaching of many of the important aspects of occlusion needed in dental practice. The term selective means the studied amount of occlusal adjustment needed to fulfil the goals of the treatment plan, e.g. from the removal of an iatrogenic occlusal interference to a comprehensive occlusal adjustment for restorative purposes. In the face of the uncertainly of time-dependent and seemingly inconclusive recommendations in qualitative systematic reviews, and the absence of an acknowledged successful therapy for refractory cases of TMD, a clinician may be faced with the studied unfortunate option of redefining evidence-based dentistry (EBD) [1], e.g., integration of [what he/she believes is] the best [available] research evidence with [their own] clinical expertise and [their own individual] patient's values.

Irrespective of the position of those in academia relative to the role of occlusal therapy in the treatment of TMDs, [2-4] the body of knowledge and practice of occlusion that is necessary for all aspects of dental care should be taught systematically in the dental curriculum [5-7].

Many of the publications dealing with OA and SS assume that every OA relates to same goal; and that SSs need only one adjustment, the one at the time of delivery. There is too often the assumption that the sample size used in research will be large enough to take into account all such variations.

### Selective Occlusal Adjustment

The teaching of OA systematically in dental schools has been nearly abandoned because of the belief that OA is an irreversible procedure and gives the impression that it is without merit elsewhere in the management of occlusion. There is no question that the initial therapy for most cases of TMD should be conservative, non-invasive therapy. Occlusal therapy and OA as the only treatment modality is not usually recommended for TMDs; however, it appears to have merit when used with other forms of therapy such as counseling, splints, and physiotherapy. [7] This essay is not an attempt to justify the use of OA for TMDs; it is a call to bring clinical reality into the dental curriculum. The uses and misuses of OA and SS and their limitations should be taught. It is not unusual to find students grinding on an opposing tooth to accommodate their newly polished gold restoration, (something that could have been avoided if the casts had been properly articulated or if they had correctly reduced the tooth relative to clearance). Unfortunately, articulation of triple tray impressions are not something the student is familiar with nor articulating casts in general. Improper occlusal clearance, on the other hand, is commonly accomplished in the region of the stamp cusp or the functioning cusp.

On boards and in clinical examinations it is found altogether too often that a mere occlusal contact on the non-working (balancing side) in mediotrusive position is called an occlusal interference. Such contact is not an occlusal interference. In order to be called a mediotrusive (non-working side) interference, the occlusal contact relation has to cause an interference with functional contact relations elsewhere, e.g., interfere with laterotrusive (working side) contacts. Some limitation of this example should be considered: a mediotrusive contact on a gold crown may become an interference problem because of the different wear rates of gold and normal enamel on the laterotrusive (working) side, especially with bruxism. To cover all the systematic teaching about occlusion that should be a part of the dental curriculum is well beyond the space that is allowed here.

### Teaching selective Occlusal Adjustment

The teaching of selective occlusal adjustment should include all the following, including clinical experience, but not limited to just the following: [9,10].

1. Systematic (structured goal outcomes) correction of occlusal contact relations that:

• interfere with function

• prevent closure into the intercuspal position

• cause excessive loading of implants

• are needed for endodontic treatment

• is needed for proper restorative treatment

• involve cracked teeth

• cause or contribute to periodontal trauma

• prevent appropriate design of splints

• enhance occlusal stability

2. Iatrogenic restorations that:

• aggravate bruxing or clenching

• immediately precede TMD-like symptoms

3. Proper emphasis on patient-centered criteria of what is perceived to be important by each patient, including shared decision-making, informed consent, and dealing with the difficult patient, e.g., phantom bite.

None of the above aspects of teaching selected occlusal adjustment is controversial; the impact of their studied absence from systematic courses in occlusion both in the literature and in the clinic has become obvious.

Some systematic and other research review papers seem to suggest that it is possible to do an occlusal adjustment in the presence of temporomandibular joint and/or muscle dysfunction. However, in order to do an occlusal adjustment how often is it possible to obtain a point of reference (CR, ICP/CO, NMP) in order to do an occlusal adjustment in the presence of significant and painful temporomandibular joint and/or muscles disorders? Without a goal and some reference position of the mandible, grinding on the teeth is not an acceptable occlusal adjustment. It is interesting that at least three textbooks on occlusion teach occlusal adjustment but where does one find it being taught systematically?

### Teaching the use of stabilization splints

The systematic teaching of the use of occlusal bite plane stabilization splints should include, but not necessarily be limited to the following: [8].

1. Diagnostic procedures

a. Diagnostic procedures required for determining the basis for use of the stabilization splint, as well as the occlusal factors that determine the design of the splint, e.g., curve of Spee, vertical overlap, extruded teeth, Angle class of malocclusion, location of TMJ clicking (opening and closing), determination of type of TMD. Shim stock should be used to determine the presence or absence of all supporting cusp contacts.

b. Patient evaluation: avoid forgetting that symptoms are the sine qua non of diagnosis; consider the patient's potential for compliance; determine if there are any problems other than "TMJ" that are of greater significance to the patient.

2. Uses of stabilization splints

a. Primary dental treatment for controlling the effects of parafunction (e.g., bruxing, clenching), and for protection against cheek and lip biting (behavioral modification), for limiting periodontal trauma from occlusion, for control of occlusal forces on implants, and for fractured teeth as secondary treatment for bruxism in cases of coexisting comormid disorders (e.g., ADD, Parkinsonism, and Bipolar disorders) where bruxism occurs.

b. Adjunctive treatment for secondary otalgia (earache) associated with clenching; subjective hearing disorder ("stuffiness") associated with some TMDs.

c. Selective treatment for symptoms of TMJ disk derangement, TMJ clicking and episodic locking, TMJ arthralgia and arthritis, myalgia, adjunctive treatment for tension-type headaches, chronic daily headache and some types of migraine [10].

3. Design of several types of stabilization splints

a. Flat plane splint that utilizes bilateral balanced occlusion to the extent possible, and incisal guidance is a constant feature.

b. Flat plane splint with canine rise but no incisal guidance. Splint designed to provide for freedom in splint centric. Canine rise varies in pitch to prevent mediotrusive contacts, laterotrusive contacts, and protrusive contacts away from splint centric

c. Generally a splint should be about 2 mm in thickness; however, the actual possible thickness may related to prevention of a closing click, sharpness of the curve of Spee, canine rise, and vertical overlap.

d. Splints should be made of heat processed acrylic.

e. Most stabilization splints utilize the maxillary arch.

4. Adjustment of stabilization splint

a. The splint should be stable with all contact movements. Retention may be obtained by undercuts (usual) or clasps (not often needed).

b. On mandibular closing all supporting cusps should make simultaneous contact in tap centric, swallowing, yawning, muscular closure and operator guided closure.

c. Adjustment of the splint takes place over a period of time consistent with mandibular repositioning due to changes in muscles and joints, as well as behavioral modifications

Every interocclusal device by whatever name it is called requires oversight adjustments to meet the individual needs of every patient. There is no occlusion or patient so "standard" that adjustment and maintenance of a splint is not needed as long as the splint is worn. It is assumed incorrectly that jaw reference positions (e.g., CR, ICP/CO, NMP) used in constructing splints are possible to determine accurately in most instances in the presence of pain and muscular dysfunction. Another assumption is that a single design, a single splint adjustment, and/or a single time period for use of a stabilization splint will "work" for all TMDs. It is possible to deduce such metamorphic thinking of "one size fits all" from many of the studies in the literature.

## Conclusion

Only two of the many aspect of the management of occlusion that need to be included more systematically in the dental curriculum have been addressed, OA and SS therapy However, there are many more that should to be considered. Hopefully, because of their importance, other aspects of the management of occlusion will once again become a significant part of the dental curriculum.

## Competing interests

The author(s) declare that they have no competing interests.
